# EuroScore 2 for identification of patients for transapical aortic valve replacement - a single center retrospective in 206 patients

**DOI:** 10.1186/1749-8090-7-89

**Published:** 2012-09-21

**Authors:** Andreas Goetzenich, Imke Deppe, Heike Schnöring, George L Gafencu, Dumitrita-Alina Gafencu, Hülya Yildirim, Lachmandath Tewarie, Jan Spillner, Ajay Moza

**Affiliations:** 1 Clinic for Cardiothoracic and Vascular Surgery, University Clinic RWTH Aachen, Pauwelsstr. 30, Aachen 52074, Germany; 2 Transplantation and Cardiac Surgery, The Royal Brompton and Harefield Trust, Harefield, United Kingdom

**Keywords:** Risk models, Transapical valve replacement, Valve disease

## Abstract

**Background:**

Operative risk scoring algorithms identify patients with severe AS for transcatheter valve implantation in whom the anticipated operative mortality for conventional surgery would be considered prohibitive. We compared the three risk scores EuroScore 1 (LES), society of thoracic surgeons’ (STS) score and ACEF (age-creatinine-ejection fraction score) to the readjusted EuroScore 2 recently presented.

**Methods:**

We reviewed all consecutive patients receiving either isolated conventional aortic valve replacement (cAVR) or transapical aortic valve implantation (TA-TAVI) in a two-year period (n = 206). 30-days mortality was considered as primary endpoint.

**Results:**

TA-TAVI was performed in 76 patients, isolated cAVR in 130 patients. Overall mortality was 4.4% (TA-TAVI: 7.9%; cAVR: 2.3%). EuroScore 2 showed a good estimation for the entire population as well as within the subgroups: 4,02 ± 5,36% (TA-TAVI: 6.16 ± 7.14%, cAVR: 2.77 ± 3.42%). Predicted mortalities as assessed by LES were largely overestimated (TA-TAVI: 27.4 ± 20.9% cAVR: 10.6 ± 10.6%, sensitivity: 0.89, specificity: 0.71). STS predicted mortality was 6.3 ± 4.4% for TA-TAVI patients as to 3.2 ± 3.1% for cAVR patients (sens.: 0.22, spec.: 0.96) and ACEF predicted a mortality of 1.16 ± 0.36% for cAVR and 1.58 ± 0.59% for TA-TAVI patients (sens.: 0.78, spec.: 0.89).

**Conclusion:**

The newly refined EuroScore 2 showed a good correlation within the studied population. For the individual patient, new cut-offs will have to be defined to triage patients for TAVI procedure. A drawback for complex score systems such as EuroScore and STS is the lack of recalibration to smaller populations as encountered in even large single centers.

## Background

Calcific Aortic Stenosis (AS) still is the most common acquired heart valve disease in western countries. [1] Aortic valve replacement (AVR) has been the gold standard for the treatment of severe AS for decades, and the indications for its implementation have been well defined by the American College of Cardiology / American Heart Association in 2006 [2] or the 2007 European Guidelines. [3] However, there is growing evidence across western countries that over 30% of patients with severe AS are not referred for AVR. Reasons given for nonoperative management included “old age”, “severe comorbidities” and “patient refusal” [4,5]. Transcatheter alternatives to standard AVR have been developed and successfully deployed in patients in whom the risk of conventional AVR (cAVR) for severe AS was considered to be too high. Operative risk scoring algorithms are currently being used to identify and select patients with severe AS for transcatheter valve implantations (TAVI) in whom the anticipated operative mortality for conventional surgery would be considered prohibitive. However, the accuracy of these risk models in identifying high-risk patients appropriate for non-standard valve therapy is not fully validated. TAVI – either transapical or transfemoral - is currently recommended in patients exceeding a logarithmic EuroScore of 20% according to recent evidence on safety and efficacy of TAVI [6]. LES, although accurate in correlating predicted and observed mortality in ranges below 5-10%, [7] has been clearly demonstrated to overestimate expected mortality by a factor of three [8] in high-risk candidates for AVR. For this cause, a recalibrated version, named EuroScore 2, was recently presented [9] and first validation studies were published [10].

The STS score system is a very complex model and difficult to obtain for all routine patients. [11,12] In the study by Dewey and Herbert [13] which compared the accuracy of the old LES and the STS in the highest risk patients, in the latter the observed mortality was much closer to the expected with an underestimation of 0.8 rather than overestimation by factor 3. STS risk algorithm, albeit not perfect, is soundly based on surgical outcome. It has clearly become the preferred method of risk assessment and evaluation. Although the predictive value of a score might be of interest, its discriminative power is of higher importance when it is used to triage patients.

Recently, the group of Ranucci et al. introduced the ACEF-score, presenting a simple score including as few as three different parameters: patient’s age, ejection fraction and serum creatinine [14]. However, various factors that also impact mortality but are not incorporated in either algorithm include liver disease, frailty, porcelain aorta, neurocognitive dysfunction, and previous radiation [15].

While the logistic EuroScore (LES) is routinely determined preoperatively in every patient admitted to our department, we performed a retrospective analysis of all consecutive patients admitted for either isolated conventional aortic valve replacement or transapical aortic valve implantation between January 2008 and December 2009. We compared the three presented risk scores ACEF, STS and LES to the recently presented readjusted EuroScore 2 as well as further details on hospitalisation and laboratory parameters.

## Methods

Since 2008, the transapical aortic valve implantation (TA-TAVI) has been performed in our department. We reviewed all consecutive patients receiving either isolated conventional av-replacement (cAVR) or TA-TAVI in a two year period, starting on January 1st 2008 until December 31st 2009 (n = 206).

Within this period, the decision for either TA-TAVI or cAVR was mainly based on the LES and made by an interdisciplinary board of cardiologists and cardiac surgeons. It was guided by the recommendations that accompanied the CoreValve safety and efficacy trials that were later included into the aforementioned recommendations, where among other criteria, a LES cut-off value of 20% was recommended.

### End points

Mortality within 30 days of the procedure was considered as primary endpoint. Several other clinical parameters were evaluated as secondary end points. These can be summarized as figures on hospitalisation (duration of operative procedures, time on respirator and days on ICU) and laboratory parameters (troponin T, CK-MB, creatinine).

### Statistical analysis

All values are given as mean and standard deviation where appropriate. Statistical analysis was performed using PASW 18 (IBM Statistics, USA) on an Apple System (Apple, USA). ANOVA or Student T-tests were performed where appropriate. Correlation analysis of Euroscore 2 vs. LES was performed by use of the Kendall-Tau-b test. P-Values are given in exact numbers; a p-value smaller 0.05 was highlighted as a statistically significant outcome. Boxplots were used to identify individual relationship between EuroScore 2 and LES, STS or ACEF. Mosaic plots were used to graphically represent the 3-dimensional contingency tables. Discriminatory power was assessed by receiver-operation characteristic-curve (ROC-curve) evaluation and the c-index (AUC; area under the ROC-curve). Cut-off values for STS and LES were derived from current therapeutic recommendations. The cut-off for the ACEF score was calculated from the point of maximal specificity and sensitivity (Youden’s Index). A cut-off value for the EuroScore 2 is still to be presented. Due to the small number of patients included in our study, a calibration was statistically not permitted for the complex EuroScore 2, yet we decided to present a cut-off based on the Youden’s Index as well to enhance comparison to the other score systems. It has to be emphasized that this cut off lacks a statistical validity.

Research carried out on humans was in compliance with the Helsinki Declaration. The study was approved by the local ethics committee and following informed consent.

## Results

Between January 1st 2008 and December 31st 2009, 206 patients were admitted for isolated aortic valve replacement. Of these, TA-TAVI was performed in 76 patients; isolated cAVR by biological or mechanical valve prosthesis was performed in 91 and 39 patients, respectively. Sex was almost evenly distributed (46.6% females in all patients) although more females received TA-TAVI (59.2%; 39.2% for cAVR). Overall mortality was 4.4%, with 7.9% in the TA-TAVI-group and 2.3% in cAVR patients. Predicted mortalities as assessed by the old logistic EuroScore were largely overestimated, namely 27.4 ± 20.9% for TA-TAVI patients compared to 10.6 ± 10.6% for cAVR patients. STS predicted mortality was 6.3 ± 4.4% for TA-TAVI patients as to 3.2 ± 3.1% for cAVR patients and ACEF predicted a mortality of 1.16 ± .36% for cAVR and 1.58 ± .59% for TA-TAVI patients. The prediction by EuroScore 2 showed a good estimation both in the entire population and within the subgroups: 4,02 ± 5,36% (TA-TAVI: 6.16 ± 7.14%, cAVR: 2.77 ± 3.42%). Further details on both patient collectives are also shown in Table 1.

**Table 1:** Demographic data of patient collective

	S**TA-TAVI**	**cAVR**	**Total**	**p between TA-TAVI and cAVR**
**Number of procedures**	76	130	206	
**Sex (% of female pts.)**	59.2	39.2	46.6	
**Age (yrs)**	79 ± 6	69 ± 11	72 ± 11	
**30 day mortality**	7.9%	2.3%	4.4%	
**Duration ofoperative procedure**	80 ± 28 min	168 ± 42 min		
**Duration on respirator**	1.5 ± 4.3 hrs	17 ± 49 hrs		**0.007**
**Days on ICU**	1.3 ± 2.9	2.9 ± 7.2		0.067
**Blood transfusions During hospital stay**	1.0 ± 2.6	3.6 ± 6.9		**0.002**
**postop CK-MB max (µg/l)**	27.1 ± 16.4	31.5 ± 27.0		0.204
**postop Trop T max (pg/ml) **	0.67 ± 0.71	0.64 ± 0.57		0.740
**preop max. creatinine (mg/dl)**	0.92 ± 0.57	0.72 ± 0.42		**0.003**
**postop max. creatinine (mg/dl)**	1.38 ± 0.84	1.05 ± 0.53		**0.001**
Δ **creatinine (mg/dl)**	0.46 ± 0.94	0.35 ± 0.61		0.312

As expected, figures on hospitalisation differ substantially between the two operative procedures. The less invasive procedure of TA-TAVI is faster (80 ± 28 min vs. 168 ± 42 min for cAVR) and results in significantly shorter duration on respirator (1.5 ± 4.3 hrs vs. 17 ± 49 hrs, p < 0.001), a substantially yet not significantly shorter ICU stay (1.3 ± 2.9 days vs. 2.9 ± 7.2 days, p = 0.067) and less blood transfusions required (1 ± 2.6 vs. 3.6 ± 6.9) during the entire postoperative period. Between the two operative procedures, no significant differences could be found for the cardiac damage markers troponin T and creatine kinase MB. The significant difference in creatinine is present pre- and postoperatively whereas its perioperative increase is similar in both treatment groups.

### EuroScore 2 and LES

**Figure 1:**
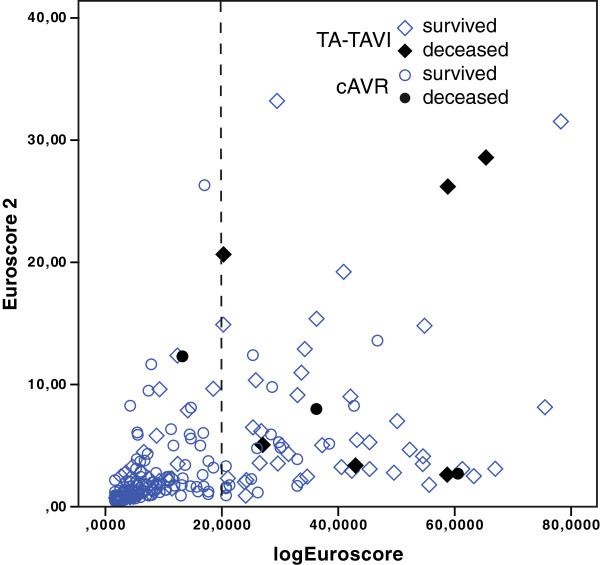
**Direct comparison of EuroScore 2 and LES. **The LES shows a wide numeric range. The cut-off value of 20% as recommended by actual guidelines is marked. Of the deceased patients in both subgroups, all but one exceeded this threshold and were preoperatively identified as high risk patients. The retrospective evaluation with the EuroScore 2 shows a poor correlation to its predecessor (τ = 0.524; p < 0.01). Only very few patients exceed a risk score of 10%.

The EuroScore 2 replaced the LES after its presentation in autumn 2011. Its acquirement is more complex, although more or less the same items are requested. The refined algorithm leads to lower predicted mortality risks in most patients, although for some cases, the new algorithm exceeds the risk predicted by LES. The correlation between the old and the new score is quite poor (Figure 1 τ = 0.524; τ_cAVR_ = 0.510; τ_TA-TAVI_ = 0.382 p < 0.001). Of course the new score will have to be validated in a large population and new cut off values to triage patients according to their perioperative risk will have to be defined. From the data collected in our study, we derived a maximal Youden’s Index at a cut-off of 2,8%. This cut-off would have identified 77 pts. as high risk, rightly including all 9 of the deceased patients.

### LES and STS score

**Figure 2:**
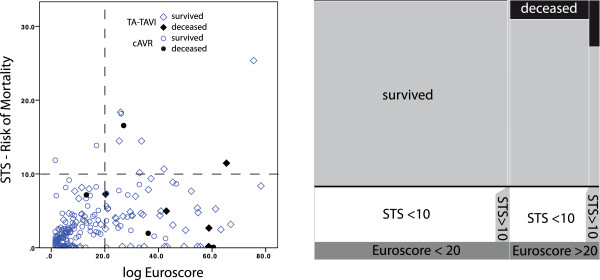
**Comparison of the STS-score to the LES in detail and as a mosaic plot. **In literature, a STS cut-off value of 10% was recommended to triage patients as “high risk”. In our population, only very few patients reach this cut off and especially the deceased patients often presented with a low STS score. In 2 high-risk patients that deceased after the operation, calculation of the STS score was impossible due to missing information. The mosaic plot is a two dimensional comparison of three variables: The size of the marked area correlates with the number of patients in the subgroup. At a glance it can be noted that only very few patients exceed an STS score of 10%, that most of these also exceed an LES of 20% and that no patient with an LES lower 20% and exceeding an STS of 10% deceased.

Whereas only 10 patients exceed a cut-off value of 10% mortality as predicted by the STS score, this holds true for 65 patients if a cut-off value of 20% mortality as predicted by the LES is taken into account Figure 2. Of these 65 patients, eight died. On the other hand, the STS score identified only 2 of the patients that died as “high risk”. So basically, the surgeons choice is between a highly specific STS-score (specificity of 0.96 in our study) that yields a very low sensitivity (0.22) or a very sensitive (0.89) LES with a fair (0.71) specificity. This is also reflected in the ROC-curve-analysis. Whereas the LES reaches an AUC of 0.837 in the entire study population (0.869 in cAVR patients and 0.727 in TA-TAVI patients), STS performance is worse: The AUC in the entire population only reaches 0.735, with 0.771 for cAVR patients and 0.561 in TA-TAVI patients. Regarding all deaths, of 9 cases only 2 were correctly marked as high risk by the STS, whereas only 1 patient was “missed” by the LES. Additionally, due to the high complexity of the score its calculation was in several cases incomplete due to missing preoperative data.

### LES and ACEF

To determine a cut-off value for the ACEF score from our study population, we observed the ROC curves and determined Youden’s index (YI = *sensitivity* + *specificity −*1) for all points on the curve. The index was maximal for an ACEF score of 1.28 both in the entire population (YI: 0.644) and in the cAVR subgroup (YI: 0.758). For TA-TAVI, the index was maximal at an ACEF score of 1.59 (YI: 0.744). In our population, maximal Youden’s index for the STS score was reached at a score of 4.75%, for the LES at a score value of 13.05%. The indices at the recommended cut-offs of 10% for the STS score and 20% for the LES were substantially lower (0.289 and 0.356, respectively), indicating a poor score performance in our study population. Due to the small population size, individual calibration of the two complex scores was statistically not permitted.

**Figure 3:**
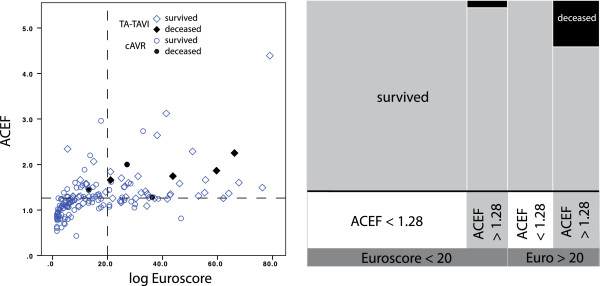
**Comparison of the calibrated ACEF to the LES. **Being the only score that can be calibrated on a small population, the ACEF can be used with an individually defined cut off for high-risk patients. The mosaic plot shows that the areas for patients with an LES < 20% and an ACEF < 1.28 are of almost even size. Whereas the LES missed to mark one patient who later died, no patient with an ACEF < 1.28 died. Most deceased were equally identified by both risk scores.

Comparison of the ACEF and the LES revealed an almost similar performance. Cut down to the ROC-AUC, ACEF reached 0.874 (LES: 0.837) for the entire population. Within the subgroups, ACEF showed an AUC of 0.871 (LES: 0.869) for cAVR patients and an AUC of 0.831 (LES: 0.727) for TA-TAVI patients. Regarding the entire study population and assuming the self defined cut-off value of 1.28, ACEF identified 61 patients as “high risk”, including all 9 deaths as compared to 65 patients identified as “high risk” by the LES including 8 of the 9 deaths (Figure 3).

## Discussion

Despite its primary conception for a specific group of patients, namely those in need of coronary artery bypass grafting [16], the LES has been widely adopted to triage patients, to identify patients at high risk or to choose the most appropriate surgical approach. It was a well known fact that the LES overestimated mortality in high risk patients. Yet the predictive value of a score system can be viewed in two different ways: The accuracy of prediction for the individual patient (the discriminative power) or the scores ability to rightly predict the overall risk of mortality for all patients (calibration). Following its recent recalibration and enhancement [9], the Euroscore 2’s capability of prediction was shown to be improved to its predecessor’s – yet whether it is still useful to classify patients as high risk has to be determined. Our study tried to evaluate the use of EuroScore 2’s discriminative power in the special case of transapical TAVI, where its predecessor was routinely used in current recommendations.

As the EuroScore 2, the STS algorithm is based on a large data set of patients collected in a current time window and the STS predictive model uses even more covariates and is therefor quite time-consuming to obtain. To be of good clinical value, a perfect score system should be easily acquired and be based on as few variables as possible. The complexity of the STS and EuroScore 2 score renders the scores less attractive for routine clinical bedside use.

Referring to “the law of Parsimony”, the ACEF Score Research Group went a step further into simplification in presenting a score that is only including three major parameters: renal function, patients’ age and the ejection fraction [17]. This simple structure is one of the scores main benefits. A more complex score requires large populations for calibration, due to the low overall mortality. A score that is defined by only three parameters can be evaluated in a small population and thus the ACEF score can be easily calibrated for each clinic, e.g. using last year’s patient collective for the upcoming year. Not only allows this a good adaptation to the treated population, changes will also reflect fluctuations within the operative statistics that can then be further evaluated.

On one hand, complex comorbidities can hardly be ignored while they present evident additional risk factors and are known to increase overall mortality and morbidity. On the other hand, the minimal influence of traditional surgical risk factors on mortality has been shown for trans-catheter valve replacement [18].

When comparing the probably over-simplistic ACEF to the detailed EuroScore2 (see also Table 2), both perform almost equally in terms of sensitivity, specificity and predictive values. As comorbidities are growing within our aging society, a detailed approach might nevertheless become necessary. To evaluate this fact, larger populations will provide a deeper insight and are surely needed to implement new recommendations into upcoming guidelines. This will have to include distinctive considerations for the different surgical and interventional approaches nowadays available.

**Table 2:** Predictive values, specificity and sensitivity of recommended or self-defined cut-offs

	**STS**	**LES**	**ACEF**	**Euroscore2**
**Cut-off**	10%	20%	1.28	2.8
**PPV**	0.20	0.12	0.15	0.17
**NPV**	0.96	0.99	**1.00**	**1.00**
**spec.**	**0.96**	0.71	0.74	0.67
**sens.**	0.22	0.89	**1.00**	**1.00**
**YI**	0.18	0.60	0.74	0.67
**F**_**1**_	0.21	0.22	0.26	0.21
**AUC**	0.735	0.837	0.874	0.853

For the STS score and the LES, predefined cut offs to identify high risk patients were taken from current recommendations. The thresholds for the ACEF and EuroScore2 score were defined from Youden’s Index after recalibration to the current data set. PPV: positive predictive value; NPV: negative predictive value; spec.:specificity; sens: sensitivity; F_1_: f-score; AUC: area under the ROC curve.

### Limitations of the study

As a single center experience, the presented data might not be representative. Yet this fact symbolizes the major problem of most average clinics: Each presents a unique patient population whose constitution is varied by many external and internal factors such as clinic size, experience but also ethnic background and the local health care system. A general comparability is desirable, yet clinical decision-making should still be the major cause for the use of risk scores. A weighed approach is therefor needed and recalibration is a strong benefit of a score.

Of course it has to be mentioned that the retrospective design of our study induced a severe bias to the perception: the performed procedure itself influences the patients’ mortality, thus, a cAVR patient would have experienced a different mortality when receiving a TA-TAVI instead. Additionally, we only included cases of transapical implantation to our study, upcoming studies will have to add evidence for the transfemoral approach.

## Conclusion

The newly refined EuroScore 2 showed a good correlation within the studied population. For the individual patient, new cut-offs will have to be defined to triage patients for TAVI procedure. A drawback for complex score systems such as EuroScore and STS is the lack of recalibration to smaller populations as encountered in even large single centers.

## Competing interests

The authors declare that they have no competing interests.

## Authors’ contributions

AG and AM designed the study, AG, HS, LT, JS and AM screened and included patients for the study, ID and HY performed the file research for STS, EuroScore 1 and ACEF, LG and DAG performed the file research for EuroScore 2. AG, JS and AM wrote the manuscript. HS and LT proofread the manuscript. All authors read and approved the final manuscript.
